# Jaagsiekte sheep retrovirus detected in human lung cancer tissue arrays

**DOI:** 10.1186/1756-0500-7-160

**Published:** 2014-03-19

**Authors:** Nicolle M Linnerth-Petrik, Scott R Walsh, Paul N Bogner, Carl Morrison, Sarah K Wootton

**Affiliations:** 1 Department of Pathobiology, Ontario Veterinary College, University of Guelph, Guelph, Ontario N1G 2W1, Canada; 2 Department of Pathology, Roswell Park Cancer Institute, Elm & Carlton Streets, Buffalo 14263, New York, USA

**Keywords:** Lung cancer, Bronchioloalveolar carcinoma, Ovine pulmonary adenocarcinoma, Jaagsiekte sheep retrovirus, Immunohistochemistry, PCR, Tissue microarray

## Abstract

**Background:**

Adenocarcinoma is the most common type of non-small cell lung cancer and is frequently observed in non-smoking patients. Adenocarcinoma in-situ (formerly referred to as bronchioloalveolar carcinoma) is a subset of lung adenocarcinoma characterized by growth along alveolar septae without evidence of stromal, vascular, or pleural invasion, that disproportionately affects never-smokers, women, and Asians. Adenocarcinoma in-situ is morphologically and histologically similar to a contagious lung neoplasm of sheep called ovine pulmonary adenocarcinoma (OPA). OPA is caused by infection with the exogenous betaretrovirus, jaagsiekte sheep retrovirus (JSRV), whose envelope protein (Env) is a potent oncogene. Several studies have reported that a proportion of human lung adenocarcinomas are immunopositive for an antigen related to the Gag protein of JSRV, however other groups have been unable to verify these observations by PCR.

**Methods:**

Here we examine human lung cancer tissue arrays (TA) for evidence of JSRV Env protein and DNA by immunohistochemical staining and PCR, respectively.

**Results:**

Our results reveal that a subset of human lung cancers express an antigen that reacts with a JSRV Env-specific monoclonal antibody in immunohistochemistry and that exogenous JSRV-like *env* and *gag* sequences can be amplified from TA tumor samples, albeit inefficiently.

**Conclusions:**

While a causative role has not been established, these data suggest that a JSRV-like virus might infect humans. With next generation sequencing approaches, a JSRV-like virus in human lung cancers may be identified which could have profound implications for prevention, diagnosis and therapy.

## Background

Lung cancer remains the leading cause of cancer related deaths worldwide with a 5-year survival rate for all stages combined of only 16% [1]. Lung cancer can be broadly divided into two groups; non-small cell lung cancer (NSCLC) and small cell lung cancer (SCLC). Pulmonary adenocarcinoma is the most prevalent NSCLC in both men and women [2]. Bronchioloalveolar carcinoma (BAC) is a subtype of pulmonary adenocarcinoma that often presents as multifocal lesions in the peripheral regions of the lung with a “ground glass” appearance by radiologic imaging. It is defined histologically by a pure lepidic growth pattern with neoplastic cells growing on preserved alveolar walls with no evidence of stromal, vascular or pleural invasion [3,4]. A recent consensus classification system for adenocarcinoma, in fact, has proposed replacement of the term BAC with “adenocarcinoma in-situ” [5]. These non-invasive lung lesions are more commonly found in non-smokers, women and the Asian population [6].

The main risk factors for lung cancer include cigarette smoke, asbestos, environmental pollution and radiation [7-10]. However, approximately 15% of all lung cancer cases are not attributable to any of these risk factors. It has been estimated that 15–25% of human cancer may have a viral etiology and two viruses in particular, the human papilloma virus (HPV) and jaagsiekte sheep retrovirus (JSRV), have been speculated to play a role in the pathogenesis of human lung cancer [11].

JSRV is the causative agent of ovine pulmonary adenocarcinoma (OPA) [12], a disease characterized by the induction of a low grade multifocal mixed adenocarcinoma with papillary and/or acinar characteristics within the distal lungs of infected sheep. OPA shares clinical and histologic features with human adenocarcinoma in-situ and is regarded as a valuable large animal model for this human disease [13-15]. The cellular receptor for JSRV is the glycosyl-phosphatidylinositol (GPI)-linked protein, hyaluronidase 2 (Hyal2) [16]. Hyal2 homologues from a variety of different species, including humans, have been shown to mediate entry of JSRV Env pseudotyped retroviral particles [17]. JSRV induces lung tumors in sheep through expression of the envelope (Env) protein [18]. Several studies have shown that the JSRV Env protein has the ability to transform a variety of cell types *in vitro *[19-21], including human cells [22], and can induce lung tumors in mice [23,24]. However, the exact mechanism by which Env mediates tumorigenesis is still unknown.

The ability of JSRV to cause peripheral lung tumors in sheep with clinical and histological similarities to those frequently found in human lung adenocarcinoma [13-15], the capacity for JSRV to use the human homolog of Hyal2 to enter human cells [16,25], and the fact that JSRV Env can transform human lung epithelial cells *in vitro *[22] has led to speculation that JSRV could be linked to a subset of human lung cancers [11]. Indeed, studies have detected JSRV capsid protein as well as *orf-x*, *gag* and endogenous JSRV sequences in human tissues [26-28]; however, there is no clear consensus on the association of JSRV with human lung cancer as other studies report no correlation between JSRV and human lung cancer [29-31]. Interestingly, a number of epidemiological studies have found that workers in abattoirs and meat processing plants have an increased risk of developing lung cancer that is postulated to be due to exposure to oncogenic viruses of food animals such as JSRV and bovine papilloma virus [32-34].

Given that JSRV Env is capable of inducing tumors in both sheep and mice, and due to the controversy surrounding the role of JSRV in human lung cancer, we decided to examine multiple types of human lung tumor samples for the presence of JSRV Env by immunohistochemical staining of lung cancer tissue arrays with an Env-specific monoclonal antibody and by PCR amplification with *env*- and *gag*-specific primers. Here we demonstrate that a subset of human lung cancers express an antigen that reacts with a JSRV Env monoclonal antibody and that *env* and *gag* sequences can reproducibly be amplified from genomic DNA extracted from human lung cancer tissue arrays, albeit inefficiently.

## Methods

### Tissue samples

Human lung cancer tissue arrays (LC2085a) containing lung tumors from 188 patients and 20 samples of normal tissue were purchased from US Biomax (Rockville, MD). Of the 208 core tissues, there were 72 adenocarcinomas, 72 squamous cell carcinomas, 22 small cell carcinomas, 2 large cell carcinomas, 10 normal lung tissues and 10 normal adjacent tissues. A human nasopharyngeal cancer tissue array (NH1001, US Biomax) containing 15 squamous cell carcinomas, 3 basal cell carcinomas, 2 adenocarcinomas, 15 papillomas, 6 polyps, 3 each of hyperplasia and inflammation and 1 adjacent normal tissue was used as a control. Note that all specimens on the lung and nasopharyngeal cancer tissue arrays were of Chinese origin. In addition, 10 examples of adenocarcinoma in-situ and 10 non-neoplastic lung tissue specimens (unstained slides and tissue cores) were obtained from formalin fixed paraffin embedded tissue blocks at the Roswell Park Cancer Institute (Buffalo, NY). Approval for the use of human tissue samples was obtained from the Roswell Park Cancer Institute. Tissue arrays were subjected to immunohistochemical analysis as described below.

### Cell lines

Human A549 cells (ATCC CRL-1573) and the ovine pulmonary adenocarcinoma cell line, JS7, (kindly provided by Dr. Mark Ackerman, Iowa State University, USA) were propagated in Dulbecco’s modified Eagle medium supplemented with 10% fetal bovine serum, 2 mM L-glutamine and 1% penicillin/streptomycin. HBE135-E6E7 cells (ATCC CRL-2741) were grown in keratinocyte-serum free medium (Invitrogen) with 5 ng/ml human recombinant EGF and 0.05 mg/ml bovine pituitary extract. Cells were maintained at 37°C in 5% CO_2_.

### Immunohistochemistry

Paraffin embedded tissue was dewaxed and rehydrated using xylene followed by decreasing concentrations of ethanol. Citrate buffer was used for antigen retrieval and tissues were blocked using 5% bovine serum albumin (BSA). Tissue sections were incubated at 4°C overnight with either a 1:50 dilution of a highly specific anti-JSRV Env monoclonal antibody [35], an isotype control antibody or supernatant from an unrelated antibody-producing hybridoma as described previously [36]. Primary antibodies were detected using a 1:50 dilution of an anti-mouse secondary antibody conjugated to biotin (Santa Cruz Technologies). Sigma Fast 3,3-diaminobenzidine tablets (Sigma, St. Louis, MO) were used to visualize protein localization in the tissue. Hemotoxylin was used as a counterstain. Tissues were analyzed by three independent observers and were graded as 0 for no staining, 1 for low intensity staining, 2 for moderate intensity staining in >10% of tissue or high intensity staining in <10% of tissue, and 3 for high intensity staining in >10% of tissue.

To confirm the specific reactivity of the anti-JSRV Env monoclonal antibody on histology sections, a peptide blocking technique was employed [37]. The peptide used in this assay was a fusion protein called JSU-IgG which is comprised of the JSRV Env surface domain (SU) fused to the human immunoglobulin G (IgG) constant region [38]. JSU-IgG was used to boost mice during the preparation of mouse hybridomas producing antibodies specific for the JSRV Env SU domain [35]. The anti-JSRV Env monoclonal antibody was neutralized by incubating with an excess of JSU-IgG (1:5 and 1:10 ratios) for 1 hour at room temperature, followed by the standard immunohistochemistry procedure described above. A JSU-IgG alone control was also performed.

### Genomic DNA extraction

Three serial sections of a human lung cancer tissue array were purchased and processed individually such that all of the tissues on one array (excluding normal tissue) were pooled together giving a total of three pooled tissue array samples, TA#1, TA#2 and TA#3, one for each array. Tissue array slides (3 in total) were first dewaxed in xylene for 30 minutes followed by rehydration of the tissues for 30 minutes each in 100% ethanol, 95% ethanol, and 70% ethanol. The slides were then washed with PBS for 15 minutes before tissue was scraped from the slides, pooled together and placed into 200 ul of PBS. Note that it was necessary to pool all of the tissue samples on the array in order to obtain sufficient genomic DNA for PCR analysis. Genomic DNA was extracted from tissue arrays, tissue cores and cell lines using the QIAamp DNA mini kit (Qiagen) in accordance with the manufacturer’s instructions.

### PCR detection of *env* and *gag* sequences

JSRV *env* and *gag* sequences were detected by PCR amplification using 100-300 ng of genomic DNA and either *env* (5′-ATACGGGAACGGATCTGGACC-3′ and 5′-CAACATGAATGGATACGGCACGC-3′) or *gag* (5′-CCCCATCTCTGAAAATGCAC-3′ and 5′-TGTTTAGACGGTGGAGGAAA-3′) specific primers which amplify 382 and 313 base pair (bp) fragments, respectively. Primers were designed according to the JSRV genomic sequence with accession number AF105220. DNA template integrity was assessed in a separate PCR reaction using GAPDH specific primers (5′-CCACCCATGGCAAATTCCATGGCA-3′ and 5′-TCTAGACGGCAGGTCAGGTCCACC-3′) which give rise to a 593 bp PCR product. For PCR amplification, the *5PRIME* MasterMix was employed and the cycling conditions were as follows: 94°C for 2 min followed by 30 cycles of 94°C for 30s, 55°C for 30s and 65°C for 2 min. A final extension of 7 min at 65°C concluded the program. PCR products were purified with the QIAquick Gel Extraction Kit (Qiagen). Purified PCR products were cloned into the pGEM-T Easy vector (Promega) following the manufacturer’s instructions and purified plasmid DNA (where possible, 3 clones per sample) was sequenced two times in both directions with an ABI Prism DNA Sequencer.

Several measures were taken to avoid contamination. All experiments were conducted at the beginning of the day in a laboratory located in a separate building that had not previously housed JSRV DNA or ovine tissue. DNA extractions and PCR amplifications were conducted using previously unopened tissue arrays, primers, DNA extraction kits, PCR amplification reagents and filtered tips.

### PCR detection of ovine genomic DNA

To test whether fragments of sheep DNA were present in the human DNA samples used in this study, a nested PCR assay was employed using published primers (mt1f; 5′-TATAGTAGGAACCGCCTTAAGCCTA-3′, mt2f; 5′-TACTCGGAGATGACCAAATCTACAACG-3′, mt3r; 5′-ATTCAGGTTTCGGTCCGTTAGTAGTATTG-3′, mt4r; 5′-TACTTCAGGGTGCCCAAAGAATCAGAATAG-3′) specific for sheep mitochondrial cytochrome-coxidase subunit 1 [27].

## Results

### Detection of JSRV Env protein in human lung cancer tissue arrays

**Figure 1:**
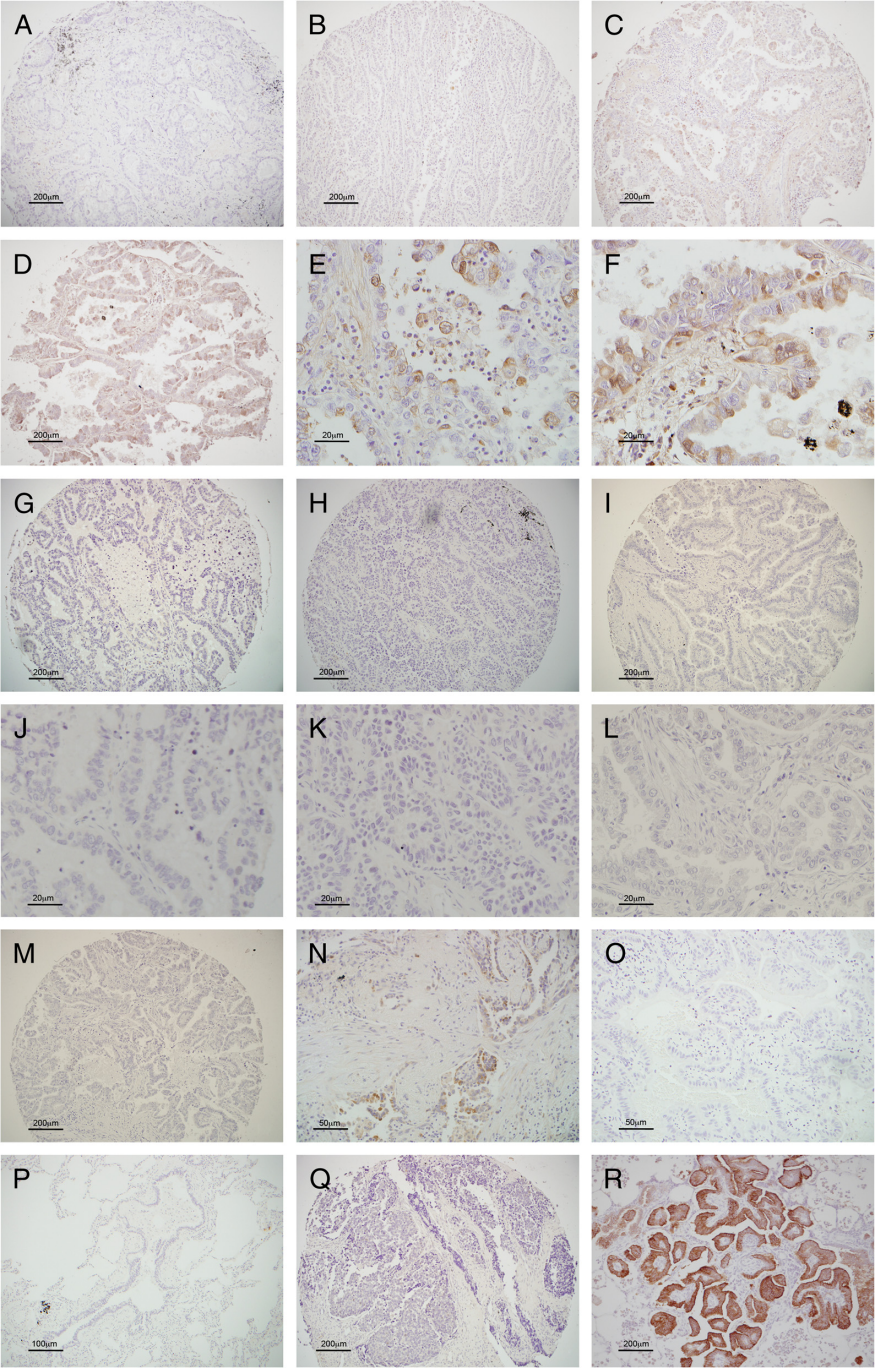
**Representative images of immunohistochemical staining of tissues with a monoclonal antibody against the JSRV Env protein.** Human lung tumor tissue array samples representing grade 0 staining **(A)**, grade 1 staining **(B)**, grade 2 staining **(C)**, and grade 3 staining **(D)**. High magnification (40x) images of human lung tumors with grade 2 **(E)** and grade 3 **(F)** staining. Low (4X) and high (40X) magnification images of human lung tumor tissue array sample stained with an isotype control (**G** and **J**) and an unrelated antibody-producing hybridoma **(H and K)**. Lack of immunohistochemical staining of human lung tumor tissue arrays in the presence of JSRV Env antibody plus purified JSU-IgG protein **(I and L)** or JSU-IgG protein alone **(M)**. Human adenocarcinoma in-situ displaying clusters of JSRV Env positive cells **(N)**. Representative images of human adenocarcinoma in-situ **(O)** and non-neoplastic human lung tissue **(P)** with no detectable Env staining. Nasopharyngeal carcinoma tissue array showing no detectable Env staining **(Q)**. Lung tissue from an OPA affected sheep stained with the JSRV Env monoclonal antibody **(R)**.

To evaluate the association between JSRV Env and human lung cancer, three sections of a human lung cancer tissue array purchased from US Biomax were analyzed by immunohistochemical staining using an anti-JSRV Env monoclonal antibody [35]. Staining, which was observed in a subset of the samples (Figure 1), was confined to epithelial cells within the tumor and varied from apical (Figure 1E) to cytoplasmic (Figure 1F). Serial sections of the lung cancer tissue array were further analyzed by immunohistochemistry using an isotype control (Figure 1G and J) as well as supernatant from an unrelated antibody-producing hybridoma (Figure 1H and K) to control for non-specific staining. In both cases, no positive staining was observed.

Staining intensity of the JSRV Env antibody was quantified by three individuals according to the grading scheme described earlier and illustrated in Figure 1A, B, C and D. Immunohistochemical staining with the JSRV Env specific monoclonal antibody yielded the following results. 21% of adenocarcinomas and squamous cell carcinomas were positive according to our criteria (grade 2 or higher), of which 2% of adenocarcinoma and 4% of squamous cell carcinomas were highly positive (grade 3) (Table 1). It should be noted that with the small number of tumor biopsies that stained highly positive (8/187 squamous cell carcinomas and 4/207 adenocarcinomas), it is unlikely that there is any statistically significant difference between adenocarcinoma and squamous cell carcinomas. Conversely, only 5% small cell carcinomas stained positive for JSRV Env. 100% of both normal tissue and adjacent normal tissue had either no staining or low intensity staining (Table 1).

**Table 1:** Total JSRV envelope staining of human lung tumor tissue arrays

	Staining intensity*			
**Lung tissue**	**0**	**1**	**2**	**3**
Adenocarcinoma	43/207 (21%)	120/207 (58%)	40/207 (19%)	4/207 (2%)
Squamous cell carcinoma	39/187 (21%)	108/187 (58%)	32/187 (17%)	8/187 (4%)
Small cell carcinoma	45/64 (70%)	16.64 (25%)	3/64 (5%)	0/64 (0%)
Adjacent normal tissue	39/46 (58%)	7/46 (15%)	0/46 (0%)	0/64 (0%)
Normal lung tissue	43/52 (83%)	9/52 (17%)	0/52 (0%)	0/52 (0%)

*Tissues were graded as 0 for no staining, 1 for low intensity staining, 2 for moderate intensity staining in >10% of tissue or high intensity staining in <10% of tissue, and 3 for high intensity staining in >10% of tissue.

The extent of JSRV Env staining was further examined in the adenocarcinoma and squamous cell carcinoma tissues with respect to stage of disease. For stage I, II and III adenocarcinoma, 17%, 21%, and 25% of tissue samples, respectively, were graded as 2 or higher (Table 2). Conversely, for stage I, II and III squamous cell carcinoma, 26%, 19%, and 20% of tissue samples, respectively, were graded as 2 or higher (Table 3). These results suggest that a subset (approximately 21%) of human lung adenocarcinoma and squamous cell carcinoma tissues express an antigen that cross-reacts with the JSRV Env-specific monoclonal antibody and that in the case of adenocarcinoma, increased staining intensity correlates with more advanced stage cancer, whereas decreased staining intensity correlates with more advanced stage cancer in the case of squamous cell carcinoma. Moreover, there was no apparent relationship between positive staining with the JSRV Env monoclonal antibody and adenocarcinomas as the small percentage of squamous cell carcinomas that stained strongly positive (4%) was twice that of strongly positive adenocarcinomas (2%).

**Table 2:** Total JSRV envelope staining in adenocarcinomas based on stage

	Staining intensity*			
**Stage**	**0**	**1**	**2**	**3**
**I**	14/54 (26%)	31/54 (57%)	8/54 (15%)	1/54 (2%)
**II**	21/93 (23%)	52/93 (56%)	18/93 (19%)	2/93 (2%)
**III**	8/60 (13%)	37/60 (62%)	14/60 (23%)	1/60 (2%)

*Tissues were graded as 0 for no staining, 1 for low intensity staining, 2 for moderate intensity staining in >10% of tissue or high intensity staining in <10% of tissue, and 3 for high intensity staining in >10% of tissue.

**Table 3:** Total JSRV envelope staining in squamous cell carcinomas based on stage

	Staining intensity*			
**Stage**	**0**	**1**	**2**	**3**
**I**	9/38 (24%)	19/38 (50%)	7/38 (18%)	3/38 (8%)
**II**	16/94 (17%)	60/94 (64%)	14/94 (15%)	4/94 (4%)
**III**	14/54 (26%)	29/54 (54%)	10/54 (18%)	1/54 (2%)

*Tissues were graded as 0 for no staining, 1 for low intensity staining, 2 for moderate intensity staining in >10% of tissue or high intensity staining in <10% of tissue, and 3 for high intensity staining in >10% of tissue.

Given the histological and clinical similarities between OPA and the adenocarcinoma in-situ form of human lung adenocarcinoma, we decided to further evaluate JSRV Env staining in ten adenocarcinoma in-situ samples and 10 non-neoplastic lung tissue samples obtained from the Roswell Park Cancer Institute in Buffalo, NY. Immunohistochemical analyses revealed positive staining in 2 out of 10 adenocarcinoma in-situ samples (Figure 1N), while all of the remaining adenocarcinoma in-situ samples (Figure 1O) and non-neoplastic tissues (Figure 1P) were negative.

Since there have been no reports of ovine betaretroviral antigen or proviral DNA detection in nasopharyngeal carcinomas from human patients, we decided to use two nasopharyngeal carcinoma tissue arrays, produced by the same company that made the lung cancer tissue arrays, as additional controls. Unlike the human lung cancer tissue arrays, there was no detectable staining in the nasopharyngeal carcinoma tissue arrays (Figure 1Q) further suggesting that the positive staining on the lung cancer tissue arrays was indeed specific.

### Amplification of JSRV *env* and *gag* sequences from genomic DNA isolated from human lung cancer tissue arrays

**Figure 2:**
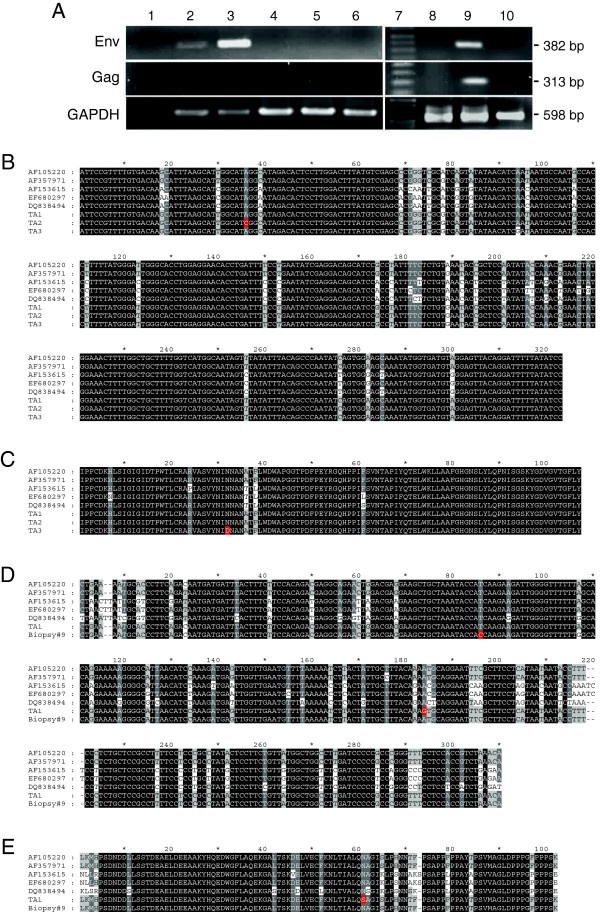
**PCR amplification of JSRV *env *and *gag *sequences from genomic DNA. (A)** Representative image of PCR products amplified using *env*- (upper panel), *gag*- (middle panel) and GAPDH-specific (lower panel) primers. Lanes 1; water only, 2; lung cancer tissue array - first round PCR, 3; lung cancer tissue array - second round PCR, 4; HBE135-E6E7, 5; A549, 6; nasopharyngeal cancer tissue array, 7; molecular weight marker, 8; non-neoplastic human lung, 9; OPA lung tumor cell line, JS7, and 10; normal sheep lung. Nucleotide **(B)** and amino acid **(C)** sequence alignment of PCR products amplified with *env*-specific primers. Nucleotide **(D)** and amino acid **(E)** sequence alignment of PCR products amplified with *gag*-specific primers. AF105220; accession number of the full-length JSRV genome deciphered by Palmarini *et al. *[12], AF357971; accession number of the full-length JSRV genome deciphered by De Martini *et al. *[39], AF153615; accession number of an endogenous JSRV genome deciphered by Palmarini *et al. *[40], EF680297 accession number of an endogenous JSRV deciphered by Arnaud *et al. *[41], DQ838494; accession number of a full-length JSRV genome of Chinese origin, TA; tissue array, Biopsy 9; adenocarcinoma in-situ #9.

To verify the immunohistochemistry results, genomic DNA was isolated from three independent serial sections of a human lung cancer tissue array and one nasopharyngeal carcinoma tissue array produced by US Biomax as well as from ten adenocarcinoma in-situ and ten non-neoplastic tissue cores. In addition, genomic DNA was extracted from two human lung tumor cell lines (A549, H358) and one immortalized human bronchial epithelial cell line (HBE135-E6E7). Genomic DNA from the OPA derived lung tumor cell line, JS7 was used as a positive control and genomic DNA isolated from ten normal sheep lungs was used as a negative control. See Table 4 for a summary of all samples analyzed. Note that aside from the OPA sample, all genomic DNA extractions and PCR reactions were conducted in a separate building detached from our laboratory to avoid the potential for contamination. Using primers specific for exogenous JSRV *env*, products of the expected size (382 bp) were amplified specifically from DNA extracted from the lung tumor tissue arrays, albeit at very low levels (representative gel; Figure 2A upper panel, lane 2). A second round of PCR resulted in a much stronger signal (Figure 2A upper panel, lane 3). No PCR products were amplified from the human cell lines, the nasopharyngeal carcinoma tissue array, the non-neoplastic tissue, or normal sheep lung even after a second round of PCR (Figure 2A upper panel, lanes 4, 5,6, 8 and 10). First round PCR products amplified from the genomic DNA of three separate lung cancer tissue arrays were cloned. Sequence analysis of three independent clones revealed that these PCR products were 99-100% identical to that of the exogenous JSRV *env* sequence deciphered by DeMartini *et al*[39]; accession number AF357971 (Figure 2B). The JSRV *env*-like sequence amplified from two out of the three tissue arrays differed by a single nucleotide located within the surface domain (Figure 2B) and in one instance, this polymorphism resulted in an amino acid change from asparagine to aspartic acid (Figure 2C). Amplification of JSRV *env* sequence from the two Roswell Park Cancer Institute adenocarcinoma in-situ tissues that were positive by immunohistochemistry (#2 and #9) was not reproducible in that only 1 out of 3 PCR reactions resulted in a product of the expected size and despite multiple attempts, these products could not be cloned (data not shown).

**Table 4:** Summary of PCR results

**Specimen**	**Number analyzed**	**Amplicon with *env *** **primers**	**Amplicon with *gag *** **primers**
Human lung cancer tissue array	3	3/3	1/3
Human nasopharyngeal carcinoma tissue array	1	0/1	0/1
Human lung tumor cell line	3	0/3	0/3^a^
Human adenocarcinoma in-situ	10	2/10^b^	1/10
Human non-neoplastic lung tissue	10	0/10	0/10
Normal sheep lung	10	0/10	0/10

^a^PCR products of the correct size were amplified but did not contain gag sequence.

^b^PCR product amplified in only 1 out of 3 attempts.

To confirm that the anti-JSRV Env antibody was specific and without cross-reactivity, additional tissue arrays were tested by peptide neutralization using purified JSU-IgG [38]. Immunohistochemistry was done as before, with the exception that slides were incubated with JSRV Env antibody + purified JSU-IgG protein or JSU-IgG protein. The positive staining seen in Figure 1D was blocked by the added JSU-IgG (Figure 1I and L) and no staining was seen with the JSU-IgG alone (Figure 1M).

Next we attempted to amplify part of the *gag* sequence from a relatively conserved region of the JSRV genome. Although DNA from all three tissue arrays and both adenocarcinoma in-situ samples produced a PCR product of the appropriate size using *gag*-specific primers, only the product from one of the three tissue arrays and one of the two adenocarcinoma in-situ samples was successfully cloned. Interestingly, while we were not able to clone *env* DNA from the adenocarcinoma in-situ samples, we were able to clone *gag* DNA. Sequence alignment with JSRV *gag* from sheep tumors identified a single nucleotide difference in the matrix region just upstream of variable region 2 [42] in both the tissue array and adenocarcinoma in-situ DNA (Figure 2D). In the case of the tissue array sequence, this nucleotide difference would result in a single amino acid substitution of an asparagine for a serine (Figure 2E). Although we observed bands of the expected size in the human lung tumor cell lines (Figure 2A middle panel, lanes 4-6), sequence analysis failed to reveal the presence of JSRV-like *gag* sequence.

### Sheep mitochondria DNA was not detected in human genomic DNA samples

**Figure 3:**
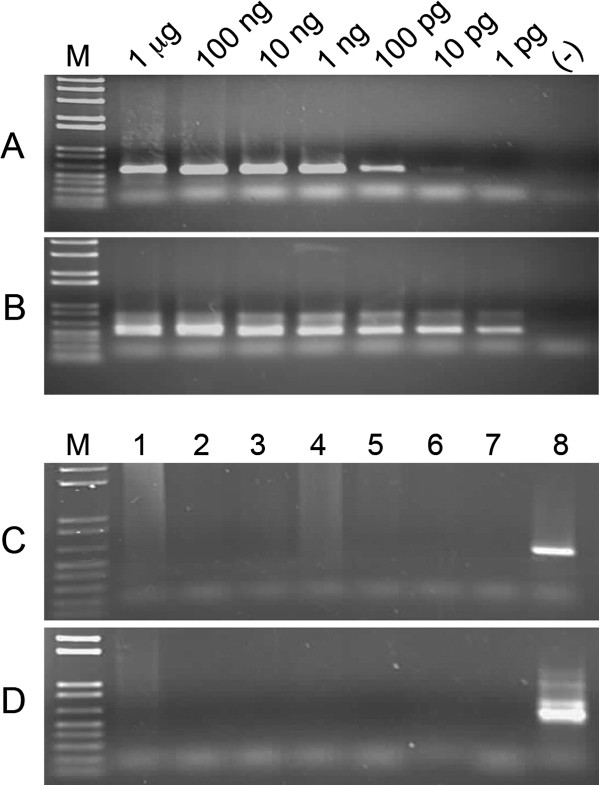
**Absence of sheep mitochondria in human DNA samples.** PCR **(A)** and nested PCR **(B)** of serial dilutions of sheep genomic DNA. First round PCR **(C)** and nested PCR **(D)** of genomic DNA isolated from non-neoplastic tissue (lane 1), lung cancer tissue array #1 (lane 2), lung cancer tissue array #2 (lane 3), lung cancer tissue array #3 (lane 4), adenocarcinoma in-situ sample #2 (lane 5), adenocarcinoma in-situ sample #9 (lane 6), water (lane 7) and the OPA lung tumor cell line, JS7 (lane 8). M; marker.

To rule out the possibility that the human genomic DNA samples that were positive for JSRV *env* and *gag* sequences were contaminated with sheep DNA, we screened human DNA samples for the presence of sheep DNA using primers specific for the sheep mitochondrial cytochrome- oxidase subunit as described previously [27]. To determine the sensitivity of the assay, serial dilutions of normal sheep lung DNA were subjected to first round PCR followed by nested PCR. The first round of PCR was able to detect sheep genomic DNA in as little as 10 pg of genomic DNA (Figure 3A) while the nested PCR increased the sensitivity to less than 1 pg of genomic DNA (Figure 3B). Although sheep mitochondrial DNA sequences were detected after the first round of PCR using 100 ng of genomic DNA isolated from the JS7 sheep lung tumor cell line (Figure 3C, lane 8), we were unable to detect any traces of sheep DNA in human DNA that tested positive for JSRV *env* or *gag* (Figure 3D, lanes 2-6), even after nested PCR.

## Discussion

Here we demonstrate that a subset of human lung adenocarcinomas, squamous cell carcinomas and bronchioloalveolar carcinomas express an antigen that reacts with a JSRV Env specific monoclonal antibody in immunohistochemistry. Further, we demonstrate amplification of JSRV *env* and *gag* sequences from genomic DNA isolated from an array of pooled human lung cancer tissue samples as well as *gag* sequences from individual adenocarcinoma in-situ samples. As with other studies, we did not find a strict correlation between adenocarcinoma in-situ and the presence of JSRV-like antigen. However, we did identify a subset of adenocarcinoma and squamous cell carcinomas that were immunopositive suggesting that this association is broader than previously thought.

While immunohistochemical studies using JSRV Gag specific antibodies consistently identify 20-30% of human lung tumor samples as immunopositive [26,27,30], the notion that a JSRV-like virus is associated with a subset of human pulmonary tumors remains controversial. In one study involving 249 human lung tumor samples, 30% of adenocarcinoma in-situs, 26% of lung adenocarcinomas and two of seven large cell carcinomas were immunopositive for JSRV capsid protein [26], but subsequent attempts to detect genomic DNA or RNA using PCR and RT-PCR failed to detect JSRV *gag* sequences in human lung tumor samples [30]. Interestingly, a study of normal blood donors from Cameroon (N = 44) and AIDS patients from Nigeria (N = 20) detected *orf-x* and *gag*-like sequences in 10% and 26% of whole blood specimens, respectively, but failed to detect JSRV-like sequences in a European cohort of 20 hemophiliacs, 15 blood donors, and 4 patients with adenocarcinoma in-situ or SCLC [27]. In our study, we employed a different approach and stained an array of human lung cancers with a highly specific monoclonal antibody against the oncogenic Env protein of JSRV, rather than Gag. The JSRV Env monoclonal antibody used in this study was produced against antigen that had been highly purified using affinity chromatography and high-performance liquid chromatography [35]. We cannot explain why our monoclonal antibody was able to detect JSRV Env-like antigens in human lung cancer specimens whereas a previous report using polyclonal antisera against JSRV Env was unsuccessful [29]; however, one possibility is that our monoclonal antibody is better at detecting denatured antigen. Interestingly, in that same study, 13/43 human lung adenocarcinomas that did not stain with antisera against JSRV Env, stained positive with both JSRV-CA and JSRV-MA antisera [29]. The fact that the JSRV Env monoclonal antibody did not cross-react with non-neoplastic lung tissue or nasopharyngeal carcinoma biopsies and only reacted strongly with a small subset of lung tumor samples suggests that the antibody is specific for an antigen present in human lung tumors. While we cannot rule out the possibility that the JSRV Env monoclonal antibody cross-reacts with an endogenous retroviral protein whose expression is upregulated in a subset of human lung tumors, our PCR data support the idea that the antigen detected by the JSRV Env monoclonal antibody is not simply a cross-reacting human protein.

It is important to note that the staining intensity and pattern in the TA differed somewhat from that of OPA (compare Figure 1F with Figure 1O). If a JSRV-like virus were to infect humans and initiate proliferation of lung epithelial cells, it is possible that the proliferating cells would be subject to immunoediting [43], which could potentially mutate Env and have an effect on the ability of the monoclonal antibody to recognize the JSRV Env-like protein. Interestingly, tumors that arise in transgenic mice expressing JSRV Env from an SPC promoter do not display uniform Env staining within the tumor and in some cases resemble the staining pattern seen in the TAs with grade 3 staining [24]. It is possible that if we had amplified the entire Env sequence we would have identified additional amino acid differences that might have resulted in modified glycosylation patterns and/or changes in epitope binding sites which could lead to decreased binding affinity of the monoclonal antibody.

In another study evaluating the presence of JSRV-like sequences in human lung tumors by PCR, geographical location was noted as an important factor. JSRV-like DNA was shown to be highly prevalent in Sardinian cases where sheep farming has been conducted for centuries and almost entirely absent in non-Sardinian cases from Campania, where sheep farming is largely non-existent [28]. Therefore, detection of JSRV-like DNA sequences in human lung tumors may be more likely in active sheep farming communities where there is an increased chance of human and sheep interaction. The same study also suggested that there could be a genetic component to determining the presence of JSRV in human populations as the isolated island population of Sardinia had higher rates of JSRV-like sequences among biopsies [28]. Interestingly, all of the human lung tumor biopsies and normal tissue included on the tissue arrays used in our study originated from China, the country with the largest sheep population worldwide (Food and Agriculture Organization of the United Nations) and where JSRV is endemic [44]. We also tested 10 adenocarcinoma in-situ and 10 normal human lung tissue samples from the USA, where OPA is uncommon. Two of the samples stained weakly positive for Env; however we were unable to consistently detect *env* by PCR. This is not surprising since the number of positively stained cells was relatively low and would have been assigned a staining intensity of 2 in accordance with our grading scheme. It is possible, therefore, that previous studies failed to identify JSRV DNA or RNA in human adenocarcinoma in-situ and lung adenocarcinoma cases because the samples were of US origin [30].

Occupational exposure to meat and live animals is a risk factor for developing lung cancer [45]. Persons who work in abattoirs where sheep, cattle, and pigs are slaughtered are exposed to livestock naturally infected with viruses that cause cancer in the animals. Included among this group of oncogenic viruses are JSRV and enzootic nasal tumor virus (ENTV). A recent study evaluating mortality and cancer incidence in two separate cohorts of workers from abattoirs (N = 4996) and meat processing plants (N = 3642), confirmed that there is an excess occurrence of cancer, particularly of the lung, in both abattoir and processing plant workers [33]. Similar results were previously reported for this and other related cohorts worldwide [34,46,47], suggesting that steps should be taken to protect workers from exposure to carcinogenic viruses in the workplace and that there could potentially be health implications for the general population which may also be exposed to some of these viruses. Interestingly, a recent report out of Europe suggests that professional exposure to goats increases the risk of pneumonic-type lung adenocarcinoma in humans [48].

## Conclusions

The association between JSRV and human lung cancer is controversial; however, we have demonstrated the presence of an antigen that cross-reacts with a monoclonal antibody specific for the JSRV Env protein in human lung tumors and have amplified *env-* and *gag-*like sequences from these samples raising the possibility that JSRV could potentially contribute to lung tumorigenesis in humans. With high-throughput next generation sequencing approaches, a JSRV-like virus in human lung cancers may be identified which could have profound implications for prevention, diagnosis and therapy.

## Competing interests

The authors declare that they have no competing interests.

## Authors’ contributions

NMLP, SRW and SKW performed the experiments. NMLP and SKW designed the studies. PNB and CM provided normal and BAC human samples and assisted with the histopathology. All authors contributed to drafting and editing the manuscript. All authors read and approved the final manuscript.
